# Lactylation in post-stroke fatigue: linking metabolic dysregulation to neuroinflammation

**DOI:** 10.3389/fnins.2025.1713149

**Published:** 2026-01-08

**Authors:** Zekai Hu, Qingui Sun, Xinhao Liu, Jinyan Wang, Xieyun Jin, Jun Hu

**Affiliations:** 1 Department of Rehabilitation Medicine, The Second Rehabilitation Hospital of Shanghai, Shanghai, China; 2 Shanghai Advanced Research Institute, Chinese Academy of Sciences (CAS), Shanghai, China; 3 College of Rehabilitation Medicine, Shanghai University of Traditional Chinese Medicine, Shanghai, China; 4 Guangming Traditional Chinese Medicine Hospital, Pudong New Area, Shanghai, China

**Keywords:** energy metabolism, inflammatory response, lactylation, neuronal apoptosis, post-stroke fatigue, protein post-translational modification

## Abstract

Lactylation, a recently identified post-translational modification derived from lactate, has emerged as a key immunometabolic regulator in neurological disorders. In the context of ischemic stroke, abnormal lactate accumulation not only reflects energy metabolism dysfunction but also drives protein lactylation, which dynamically influences neuronal survival, glial activation, and neuroinflammatory cascades. Increasing evidence indicates that lactylation modulates transcriptional programs of microglia and astrocytes, amplifying inflammatory responses through histone modifications and metabolic enzyme regulation. These processes contribute critically to the onset and persistence of post-stroke fatigue (PSF), a debilitating complication that impairs recovery and quality of life in stroke survivors. This review integrates recent findings on lactylation-mediated regulation of immune and inflammatory pathways, with a particular focus on its effects on apoptosis-related signaling, mitochondrial dysfunction, and cytokine production. Furthermore, we highlight lactylation-related enzymes, including p300 and HDAC3, as potential therapeutic targets, and discuss emerging biomarkers for monitoring lactylation dynamics in stroke patients. By framing lactylation as a metabolic–epigenetic bridge connecting cellular energy states with immune responses, this article provides new insights into the immunopathogenesis of PSF and identifies promising directions for translational intervention.

## 1 Introduction

Post-stroke fatigue (PSF) is a prevalent and debilitating syndrome that significantly impairs the rehabilitation process and quality of life in stroke survivors. The multifactorial nature of PSF encompasses disturbances in energy metabolism, neuroinflammatory processes, neuronal injury, and repair mechanisms, reflecting the complex pathophysiology of the post-stroke brain. Clinical and experimental evidence indicates that patients frequently experience persistent mental and physical exhaustion, often accompanied by psychological and emotional distress, which collectively hinder recovery and reintegration into daily activities (Panossian et al., 2025). The intricate interplay between disrupted neurotransmission, imbalance in the glutamate-glutamine cycle, alterations in glucose metabolism, and compromised ATP supply underpins the manifestation of PSF. Furthermore, the process of neural regeneration and functional restoration after stroke involves dynamic crosstalk among neuronal, glial, immune, and endothelial cells, orchestrating neurogenesis and angiogenesis, yet often failing to fully restore homeostasis (Panossian et al., 2025). These insights underscore the urgent need to elucidate the molecular underpinnings of PSF and to identify novel therapeutic targets that can mitigate fatigue and promote neurological recovery.

Traditionally, lactate has been considered a mere metabolic byproduct of anaerobic glycolysis and a contributor to muscle and brain fatigue. However, recent advances have redefined lactate as a multifaceted molecule with both beneficial and detrimental roles in human health and disease (Kumar et al., 2025). In the context of neurological disorders, including ischemic stroke, lactate serves not only as an essential energy substrate—capable of supporting approximately 8–10% of cerebral energy metabolism at rest, and up to 20–25% during increased activity (Pedersen et al., 2025). Notably, excessive accumulation of lactate after stroke is associated with tissue damage, metabolic derangements, and exacerbation of neurological deficits, while moderate increases may facilitate neuroprotection and recovery under certain conditions (Kumar et al., 2025; Pedersen et al., 2025). The duality of lactate’s effects in the brain has prompted investigations into the molecular mechanisms through which lactate influences neuronal fate, glial function, and neuroinflammatory responses. Central to these discoveries is the emerging concept of lactylation, a novel post-translational modification in which lactate-derived groups are covalently attached to lysine residues of proteins, thereby modulating their function, stability, and interactions (Gu et al., 2025; Wang et al., 2024).

Lactylation, particularly lysine lactylation (Kla), has garnered attention for its regulatory role in the pathophysiology of central nervous system diseases, including acute ischemic stroke and its sequelae (Gu et al., 2025; Wang et al., 2024). Experimental models of cerebral ischemia have demonstrated significant upregulation of protein lactylation in brain tissues following stroke, implicating this modification in key pathological processes such as neuronal apoptosis, neuroinflammation, and disruption of cellular energy homeostasis (Sun et al., 2025; He et al., 2024; Zhou et al., 2025). For example, proteomic analyses reveal that post-stroke lactylation affects a broad spectrum of both histone and non-histone proteins, influencing gene transcription, mitochondrial function, and cell survival (Zhou et al., 2025; Yao et al., 2023). Specific lactylation events, such as the modification of transcriptional regulators or metabolic enzymes, have been shown to either exacerbate or mitigate neuronal injury, depending on the context and the balance of lactate production and utilization (Sun et al., 2025; He et al., 2024). The enzymes responsible for writing (e.g., p300), erasing, and reading lactyl marks have emerged as critical mediators of these effects, offering potential targets for pharmacological intervention (Gu et al., 2025; Wang et al., 2024).

Recent studies have begun to unravel the intricate connections between lactylation and the clinical features of PSF. Elevated lactate levels and enhanced protein lactylation in the post-ischemic brain have been linked to increased neuronal death, glial activation, and persistent neuroinflammation—all of which contribute to the chronic fatigue experienced by stroke survivors (Xiong et al., 2024; Si et al., 2024). Notably, astrocyte-derived lactate has been implicated in aggravating brain injury by promoting protein lactylation and subsequent neuronal apoptosis, while pharmacological inhibition of lactate production or lactylation can alleviate these detrimental effects and improve functional outcomes (Zhou et al., 2025; Xiong et al., 2024). Moreover, lactylation-driven modulation of key proteins involved in energy metabolism, cell death pathways, and inflammatory signaling underscores its central role in the molecular pathogenesis of PSF (Sun et al., 2025; Yao et al., 2023). These findings highlight the therapeutic potential of targeting lactate metabolism and lactylation pathways to ameliorate PSF and enhance neurological recovery.

In summary, the discovery of lactylation as a dynamic and reversible post-translational modification has revolutionized our understanding of lactate’s role in the brain, shifting the paradigm from a simple metabolic byproduct to a sophisticated regulator of protein function and cellular signaling in health and disease (Gu et al., 2025; Liu et al., 2024). In the context of stroke and its aftermath, lactylation emerges as a pivotal mediator linking metabolic disturbances to neuroinflammation, neuronal injury, and the persistent fatigue that plagues many survivors. This review will comprehensively examine the current evidence on the role and mechanisms of lactylation in PSF, with a particular focus on key lactylated proteins, regulatory enzymes, and their impact on neural function and recovery. By elucidating these molecular pathways, we aim to provide a foundation for future basic and clinical research, ultimately paving the way for novel therapeutic strategies to combat PSF and improve patient outcomes.

## 2 Mechanistic insights into lactylation in post-stroke fatigue

### 2.1 Abnormal lactate metabolism and changes in lactylation levels after stroke

**Figure 1:**
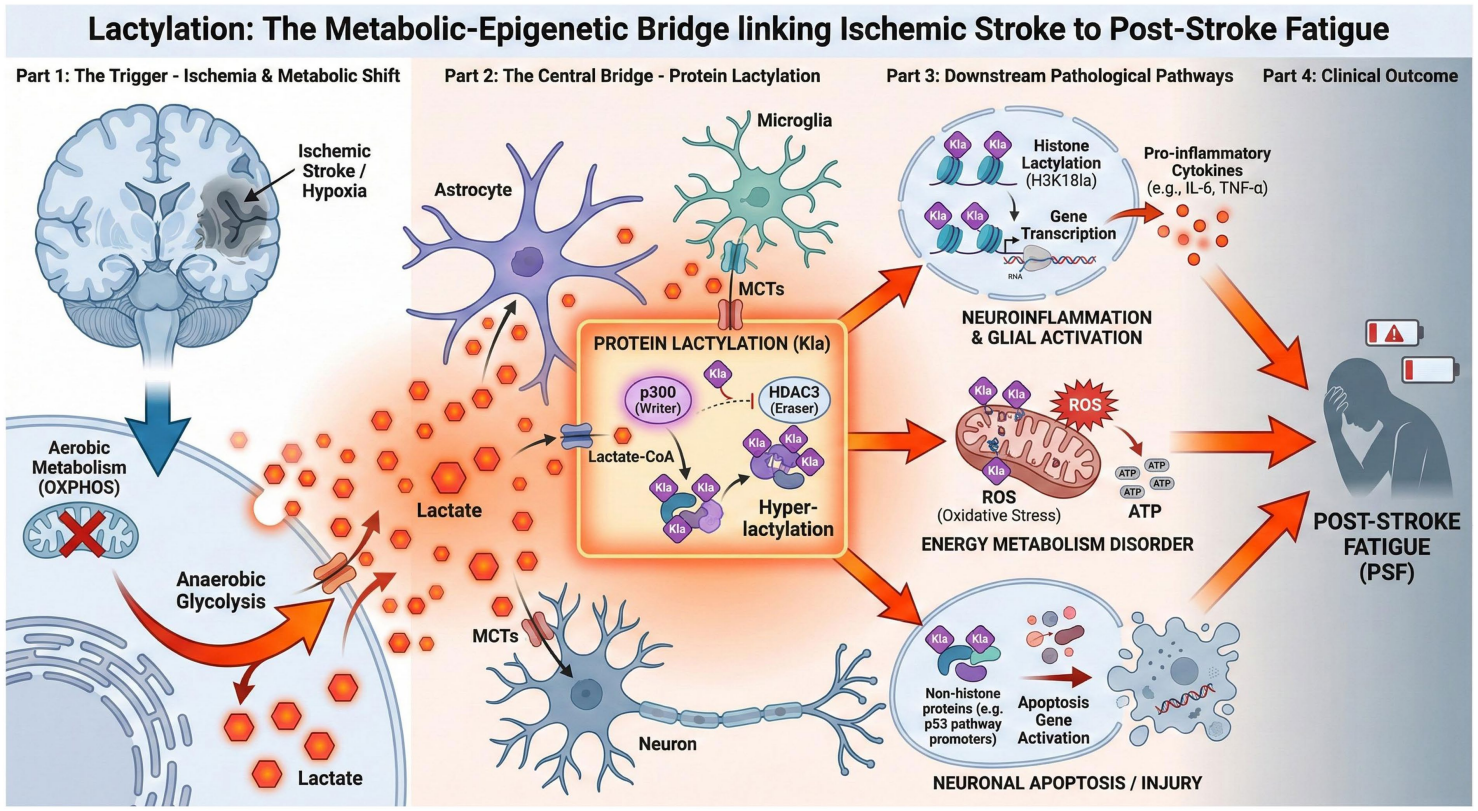
Mechanism of lactylation-mediated post-stroke fatigue. This diagram illustrates the pathological cascade linking ischemic stroke to fatigue. Part 1: Ischemia and hypoxia trigger anaerobic glycolysis, leading to massive lactate accumulation. Part 2: Accumulated lactate serves as a substrate for p300-mediated protein lactylation (Kla), shifting the cellular state from acetylation to hyper-lactylation. Part 3: Lactylation affects downstream pathways: (Top) Histone lactylation (H3K18la) promotes gene transcription of pro-inflammatory cytokines in microglia; (Bottom) Non-histone lactylation modifies metabolic enzymes and apoptosis regulators, causing mitochondrial dysfunction and neuronal apoptosis. Part 4: The cumulative effect of neuroinflammation and energy metabolism disorder leads to the clinical manifestation of post-stroke fatigue.

Following cerebral ischemia and hypoxia, the brain’s energy metabolism undergoes a dramatic shift due to a sudden decrease in oxygen and nutrient supply. Under these conditions, anaerobic glycolysis becomes the predominant pathway for ATP production, leading to a marked increase in lactate generation within brain tissue. This metabolic adaptation is primarily driven by the upregulation of glycolytic enzymes and transporters, often orchestrated by hypoxia-inducible factors (HIFs), which promote the conversion of glucose to pyruvate and subsequently to lactate via lactate dehydrogenase (LDH) (Pu et al., 2024; Madai et al., 2024). Astrocytes, which serve as the main glycogen storage cells in the brain, play a pivotal role in this process. During ischemic stress, astrocytic glycolysis is enhanced, resulting in the production and release of lactate. This lactate is then transported to neurons through the astrocyte-neuron lactate shuttle (ANLS), a process mediated by monocarboxylate transporters (MCTs), particularly MCT1 and MCT4 in astrocytes and MCT2 in neurons (Yamagata, 2022; Kang et al., 2023). After stroke, this shuttle becomes abnormally active, as the demand for alternative energy substrates in neurons rises sharply due to impaired oxidative phosphorylation. However, the excessive accumulation of lactate, especially when its production outpaces clearance or utilization, leads to tissue acidosis and can exacerbate neuronal injury by promoting protein lactylation, neuroinflammation, and cell death (Xiong et al., 2024; Kuang et al., 2024; Liao et al., 2025). Additionally, the disruption of the balance between astrocytic lactate production and neuronal uptake—whether due to impaired transporter function, altered enzyme expression, or glial dysfunction—further contributes to pathological lactate buildup (Yamagata, 2022; Zhou et al., 2024). Notably, experimental studies have shown that pharmacological inhibition of lactate production or blockade of the lactate shuttle can attenuate ischemic brain injury, underscoring the dual-edged nature of lactate metabolism in the post-stroke brain (Xiong et al., 2024). Thus, the mechanisms of lactate accumulation after stroke encompass not only the metabolic shift toward glycolysis and enhanced astrocyte-neuron lactate transfer but also the dysregulation of these processes, which collectively contribute to the pathophysiology of post-stroke brain damage (Figure 1).

Following this pathological accumulation, lactate functions not merely as a metabolic byproduct but as a substrate for a specific post-translational modification. Lactylation is a recently discovered post-translational modification in which a lactyl group, derived from lactate, is covalently attached to the *ε*-amino group of lysine residues on proteins, forming lysine lactylation (Kla) (Gonzatti et al., 2025). The process of protein lactylation can occur via both enzymatic and non-enzymatic mechanisms. Enzymatically, specific “writer” enzymes such as p300/CBP have been shown to transfer lactyl groups from lactyl-CoA to lysine residues on substrate proteins, while other studies have identified additional enzymes like AARS1 and KAT8 as lactyltransferases (Gonzatti et al., 2025; Zong et al., 2024; Xie et al., 2024). Non-enzymatic lactylation can also occur through reactive intermediates such as S-D-lactoylglutathione, particularly under conditions of elevated intracellular lactate, such as hypoxia or enhanced glycolysis (Gonzatti et al., 2025; Iozzo et al., 2025). The modification is reversible, with “eraser” enzymes such as histone deacetylases (HDACs) and sirtuins capable of removing lactyl groups, thereby dynamically regulating the lactylation status of proteins in response to metabolic cues (Gonzatti et al., 2025; Sun et al., 2022; Zhang et al., 2025).

**Figure 2:**
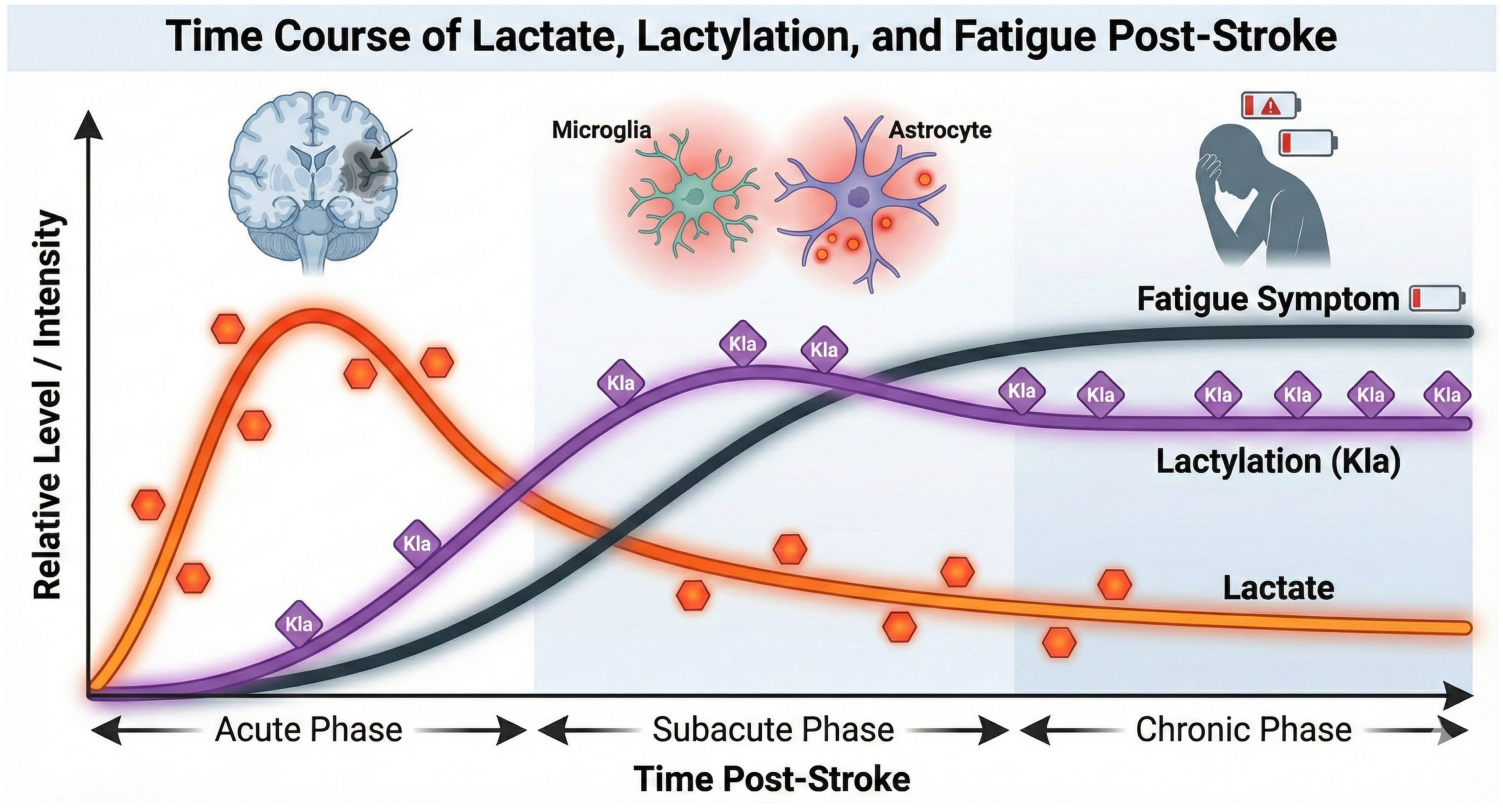
Temporal dynamics of lactate, lactylation, and fatigue after stroke. A schematic representation of the time course of pathological changes. Acute phase: Lactate levels surge immediately after stroke, followed by a rapid rise in protein lactylation. Subacute to chronic phase: While lactate levels may decrease, lactylation modifications can persist or remain elevated due to enzymatic “writing,” contributing to sustained glial activation and metabolic depression. This persistent molecular alteration parallels the development and chronicity of fatigue symptoms in the later stages of recovery.

Building on the understanding of its formation, it is crucial to examine the temporal dynamics of this modification in the ischemic brain. Dynamic changes in lactylation levels, particularly those involving lactate metabolism, have been closely observed in both animal models and human studies of stroke, revealing a distinct temporal pattern that reflects underlying pathophysiological processes. In the acute phase of ischemic stroke, there is a pronounced elevation in lactate concentration. Clinically, relevant data derived from advanced neuroimaging techniques, such as high-resolution three-dimensional magnetic resonance spectroscopic imaging (MRSI), have provided direct evidence of this metabolic shift. Studies have consistently demonstrated significantly increased lactate signals in stroke lesions during the hyperacute (0–24 h) and acute (24 h–7 days) phases (Meng et al., 2023; Li et al., 2020; Lin et al., 2023). These clinical imaging findings correlate strongly with lesion volume and tissue hypoperfusion, serving as critical biomarkers for infarction progression (Lin et al., 2023). Crucially, this clinically observed accumulation of lactate provides the necessary substrate for the subsequent surge in protein lactylation, motivating the exploration of its downstream pathophysiological effects. Animal studies utilizing rat brain slices subjected to ischemic insult further confirm this acute surge in lactate, showing a marked increase in lactate dehydrogenase (LDH) activity and a corresponding decrease in pyruvate dehydrogenase (PDH) activity, which together reflect the metabolic shift toward lactate production (Shaul et al., 2021). As the stroke evolves into the subacute and chronic phases, lactate levels may remain elevated or even increase in certain brain regions, such as the ipsilesional thalamus, indicating ongoing injury or metabolic depression (Mazibuko et al., 2020). Notably, the dynamic trajectory of lactate concentration—particularly a slower reduction over time—has been identified as an adverse prognostic indicator, with patients exhibiting persistently elevated lactate levels facing higher mortality and poorer functional recovery (Zhao et al., 2025). These findings collectively underscore the importance of monitoring lactylation dynamics as both a reflection of acute ischemic injury and a potential guide for prognosis and therapeutic intervention in stroke recovery (Figure 2).

### 2.2 Key proteins involved in lactylation regulation and their functions

#### 2.2.1 MeCP2 lactylation as a protective mechanism in stroke

Recent studies have highlighted the significant neuroprotective role of MeCP2 lactylation in the context of ischemic stroke. Specifically, the lactylation of MeCP2 at lysine residues 210 and 249 has been identified as a crucial epigenetic modification that mitigates neuronal apoptosis following cerebral ischemia (Meng et al., 2022). In a mouse model of transient middle cerebral artery occlusion, researchers observed elevated brain lactate levels and a corresponding increase in global protein lactylation within the ischemic penumbra. Proteomic analyses further revealed that non-histone proteins, particularly MeCP2, undergo significant lactylation in this region (Sun et al., 2025). Mechanistically, lactylation of MeCP2 at K210 and K249 represses the transcription of apoptosis-associated genes, such as Pdcd4 and Pla2g6, ultimately reducing the extent of neuronal cell death (Li and Cao, 2024). The protective nature of this modification is underscored by findings that chemical or genetic inhibition of MeCP2 lactylation leads to increased infarct volumes and exacerbated neurological deficits, indicating that MeCP2 lactylation functions as an endogenous defense mechanism against stroke-induced neuronal damage (Sun et al., 2025). Additionally, the regulation of MeCP2 lactylation post-stroke is mediated by key enzymes, including HDAC3 and p300, which further highlights the complexity and therapeutic potential of this pathway (Sun et al., 2025; Mi et al., 2025). Collectively, these findings suggest that MeCP2 lactylation not only serves as a molecular brake on apoptotic gene expression but also represents a promising target for interventions aimed at reducing neuronal loss and improving outcomes following stroke.

#### 2.2.2 Functional regulation of non-histone proteins in ischemic injury

Protein lactylation, initially characterized on histones, has since been recognized as a widespread post-translational modification (PTM) affecting a broad array of non-histone proteins with diverse cellular functions, particularly in the context of metabolic and signaling pathways relevant to post-stroke cellular responses. Recent proteomic studies have demonstrated that lactylation is prevalent on metabolic enzymes, such as those involved in glycolysis, including ALDOA and DHRS7, where site-specific lactylation can modulate enzymatic activity and potentially create feedback loops that fine-tune cellular metabolism in response to changing lactate levels (Wan et al., 2022). Expanding beyond metabolism, lactylation has been detected on non-histone proteins implicated in signal transduction, cell fate determination, and stress responses, highlighting its regulatory versatility (Zhao et al., 2025). Similarly, lactylation of nuclear receptor coactivator 4 (NCOA4) at K450 enhances its stability and facilitates ferritinophagy and glycolysis in neurons, contributing to exacerbated ischemic damage (He et al., 2024). Other proteins, such as LCP1 and PLBD1, are upregulated and hyper-lactylated following ischemic insult, leading to increased protein stability and activity that promote neuronal apoptosis and brain injury, effects reversible by inhibiting glycolysis or lactylation (Zhou et al., 2025; Zhang et al., 2023). Furthermore, lactylation of ARF1 in astrocytes, modulated by LRP1, influences mitochondrial transfer to neurons and impacts recovery after stroke (Zhou et al., 2024). These findings collectively underscore that non-histone protein lactylation is not only a marker of metabolic reprogramming but also an active participant in the regulation of cellular signaling, apoptosis, inflammation, and neuroprotection after stroke. The broad distribution and functional impact of non-histone lactylation suggest that it modulates stroke pathophysiology through intricate control of protein activity, stability, and interaction networks, offering novel therapeutic targets for modulating post-stroke cellular outcomes (Gu et al., 2025; Liao et al., 2025; Zhao et al., 2025). Crucially, the cumulative dysfunction of these metabolic and signaling proteins contributes to the failure of neuronal energy homeostasis. This cellular ‘energy crisis,’ driven by aberrant lactylation, manifests clinically as the profound physical and cognitive exhaustion observed in PSF patients. A summary of key lactylated proteins and their regulatory mechanisms in the context of stroke and neural injury is provided in Table 1.

**Table 1:** Key lactylated proteins and their pathological roles in stroke and neural injury.

Target protein	Protein type	Lactylation site (if identified)	Regulatory enzyme (writer/eraser)	Functional mechanism	Role in stroke/Pathological outcome	Ref.
MeCP2	Transcription regulator	K210, K249	p300/HDAC3	Represses transcription of Pdcd4 and Pla2g6	Protective: Inhibits neuronal apoptosis and reduces infarct volume	Sun et al. (2025) and Li and Cao (2024)
Histone H3	Histone	H3K18	p300/–	Promotes transcription of Nsun2 or cytokines	Detrimental: Amplifies neuroinflammation in microglia and astrocytes	Si et al. (2024) and Wang et al. (2025)
Histone H3	Histone	H3K9	–/SMEK1 (modulator)	Activates Ldha and Hif-1α transcription	Detrimental: Promotes M1 microglial polarization and inflammation	Si et al. (2024) and He et al. (2024)
NCOA4	Nuclear receptor coactivator	K450	–	Enhances protein stability; promotes ferritinophagy	Detrimental: Exacerbates neuronal oxidative damage and injury	He et al. (2024)
LCP1	Actin-binding protein	–	–	Increases protein stability	Detrimental: Promotes neuronal apoptosis	Zhang et al. (2023)
PLBD1	Phospholipase	–	–	Enhances protein stability	Detrimental: Facilitates brain injury progression	Zhou et al. (2025)
ARRB1	Scaffolding protein	–	p300	Upregulates S100A9 expression	Detrimental: Induces mitochondrial dysfunction and neuronal apoptosis	Mi et al. (2025)
MDH2	Metabolic enzyme	–	–	Alters TCA cycle flux; enhances NADPH generation	Context-dependent: Regulates mitochondrial bioenergetics and ROS	Tang et al. (2025)
ALDOA	Metabolic enzyme	–	–	Inhibits enzymatic activity	Regulatory: Modulates glycolytic flux	Wan et al. (2022)

### 2.3 Lactylation regulatory enzymes and their roles after stroke

p300, a well-characterized acetyltransferase, has emerged as a pivotal enzyme mediating lysine lactylation, thereby functioning as a “writer” of protein lactylation. This enzyme catalyzes the transfer of lactyl groups from lactyl-CoA to lysine residues on both histone and non-histone proteins, facilitating a key link between cellular metabolic status and epigenetic regulation. Given that p300 was originally identified as a histone acetyltransferase (HAT), there exists a competitive relationship between acetylation and lactylation for lysine residues. The balance between these two modifications is dynamically regulated by the intracellular concentrations of their respective substrates: acetyl-CoA and lactyl-CoA. Under physiological conditions where aerobic respiration dominates, acetyl-CoA levels are relatively high, favoring acetylation. However, in the context of ischemic stroke, the metabolic shift toward anaerobic glycolysis leads to a surge in lactate and lactyl-CoA production, while acetyl-CoA levels may decline due to impaired mitochondrial function. This metabolic reprogramming creates a cellular environment that promotes p300-mediated lactylation over acetylation, effectively coupling the cell’s metabolic state to its epigenetic landscape. Numerous studies have demonstrated that p300 is essential for the formation of protein lactylation, as evidenced by its ability to catalyze lactylation at specific lysine sites on substrates such as histones (e.g., H3K9, H3K18, H4K12) and various non-histone proteins, including transcription factors and metabolic regulators (Deng et al., 2025; Liu et al., 2025; Zhang et al., 2025). The regulatory significance of p300-mediated lactylation is underscored in pathological contexts such as ischemic brain injury, where increased lactate production leads to elevated lactylation levels, which in turn modulate gene expression and cellular fate. Recent research in models of cerebral ischemia and subarachnoid hemorrhage has shown that inhibition of p300, either through genetic knockdown or pharmacological agents such as A-485, results in a marked reduction in lactylation levels, decreased neuronal apoptosis, and attenuated glial activation (Sun et al., 2025; Mi et al., 2025). Mechanistically, p300-driven lactylation represses transcription of apoptosis-associated genes, thereby exerting neuroprotective effects following stroke. Furthermore, p300 inhibition has been shown to mitigate lactylation-dependent activation of inflammatory pathways in microglia and other glial cells, reducing neuroinflammation and subsequent neuronal damage (He et al., 2024; Qin et al., 2025). These findings collectively highlight the central role of p300 as a lactylation transferase in the pathogenesis of stroke and its sequelae, including PSF. Therapeutically, targeting p300 with specific inhibitors offers a promising strategy to modulate aberrant lactylation, protect neuronal integrity, and suppress maladaptive glial responses in the aftermath of cerebral ischemia (Lan et al., 2025). Beyond p300, other members of the histone acetyltransferase (HAT) family also play significant roles in regulating lactylation. CBP (CREB-binding protein), a close paralog of p300, shares high structural homology and often functions redundantly with p300 to catalyze histone lactylation, particularly in the context of regulating gene expression during metabolic stress (Gu et al., 2025; Liu et al., 2024). Furthermore, recent studies have identified other HATs, such as KAT8 (MOF), as capable of transferring lactyl groups to non-histone proteins, thereby influencing protein synthesis and cell proliferation (Xie et al., 2024). While the specific role of KAT8 in ischemic stroke remains to be fully elucidated, its function in cellular stress responses suggests it may serve as an additional regulatory node in the post-stroke lactylation network, warranting further investigation.

In contrast to the ‘writer’ activity of p300, the reversal of this modification is governed by specific ‘eraser’ enzymes. Histone deacetylase 3 (HDAC3) has emerged as a pivotal “eraser” enzyme capable of removing lactylation modifications from proteins, thus playing a crucial role in regulating the homeostasis of protein lactylation after stroke. Recent studies have identified HDAC3 as a histone lysine delactylase, directly involved in the dynamic regulation of protein lactylation in various pathological contexts, including cerebral ischemia, cancer, and cardiovascular diseases. For instance, in a mouse model of ischemic stroke, HDAC3 was shown to regulate the lactylation status of MeCP2, a transcriptional regulator whose lactylation at specific lysine residues represses the transcription of pro-apoptotic genes, thereby conferring neuroprotection. Inhibition or genetic manipulation that disrupts HDAC3-mediated delactylation leads to aggravated neuronal injury and larger infarct volumes, underscoring the enzyme’s importance in maintaining lactylation-mediated protective responses in the post-stroke brain (Sun et al., 2025). Beyond the central nervous system, HDAC3 also modulates lactylation in non-histone proteins, such as NBS1, where its delactylase activity is essential for efficient DNA repair and cellular resistance to chemotherapy, highlighting the enzyme’s broader biological relevance (Chen et al., 2024). Mechanistically, HDAC3 operates in concert with other chromatin modifiers, such as p300, to fine-tune the lactylation landscape in response to metabolic and environmental cues, including changes in lactate concentration after ischemic injury (Sun et al., 2025). In cardiovascular and inflammatory diseases, downregulation of HDAC3 leads to increased histone lactylation, particularly at sites such as H4K12, which influences gene expression programs related to cellular senescence, inflammation, and tissue remodeling (Li et al., 2024). Furthermore, the regulation of HDAC3 itself is subject to upstream control by factors like METTL7B, which modulates HDAC3 stability and, consequently, lactylation levels during cardiac remodeling (Chen et al., 2025). Collectively, these findings position HDAC3 as a central regulator of lactylation homeostasis, with significant implications for neuronal survival, inflammation, and tissue recovery following stroke and other pathological insults.

Beyond the actions of individual enzymes, the broader regulatory networks involving p300 and HDAC3 are intimately linked to the clinical manifestations of stroke recovery. The connection between enzyme regulatory networks and PSF is increasingly recognized as a critical aspect of the pathophysiology underlying fatigue after cerebral ischemic events. Enzymes involved in lactylation and related metabolic pathways play a pivotal role in modulating energy metabolism and neuroinflammation, both of which are central to the development and persistence of fatigue symptoms. For instance, changes in the expression and activity of key metabolic enzymes can disrupt the balance of energy production and consumption in neural tissues, exacerbating fatigue. Furthermore, inflammatory enzyme networks, including those regulating the production of cytokines like interleukin-6 and high-sensitivity C-reactive protein (hs-CRP), have been shown to correlate with the severity of fatigue in stroke survivors. However, while some studies report associations between these inflammatory markers and fatigue scores, these relationships may not remain significant after adjustment for confounding variables (Gyawali et al., 2020; Liu et al., 2020). Additionally, enzymes such as glucose-6-phosphate dehydrogenase (G6PD) are implicated in redox balance and energy homeostasis; deficiencies or dysfunctions in these enzymes can lead to metabolic disturbances manifesting as fatigue, especially in the context of stroke and its treatments (Li et al., 2024). Collectively, these findings underscore the multifaceted role of enzyme regulatory networks in orchestrating the metabolic and inflammatory processes that contribute to PSF, highlighting the potential for targeted modulation of these enzymes as a therapeutic strategy to alleviate fatigue and improve patient outcomes.

### 2.4 The relationship between lactylation, neuronal apoptosis, and post-stroke fatigue

#### 2.4.1 Lactylation and neuronal apoptosis

While metabolic dysregulation and lactate accumulation constitute the initial biochemical insult following stroke, their pathological consequences are ultimately realized through downstream cellular events, most notably neuronal apoptosis. Lactylation serves as a critical mechanistic link converting this metabolic stress into a regulated cell death program. As energy failure compromises neuronal viability, the concomitant surge in lactylation modifies key apoptotic regulators, thereby determining neuronal fate. Recent advances have elucidated the multifaceted role of lactylation—a post-translational modification derived from lactate—in the regulation of neuronal apoptosis, particularly in the context of brain injury and neurodegenerative processes. Notably, lactylation exerts its regulatory influence by modulating the transcription of apoptosis-related genes, thereby impacting neuronal survival. For instance, in models of ischemic stroke, lysine lactylation of the transcriptional regulator MeCP2 at specific sites (K210/K249) has been shown to repress the transcription of pro-apoptotic genes such as Pdcd4 and Pla2g6, effectively attenuating neuronal apoptosis and conferring neuroprotection. Inhibition of MeCP2 lactylation, conversely, leads to increased infarct volume and worsened neurological outcomes, highlighting the protective capacity of this modification. The regulation of MeCP2 lactylation is orchestrated by epigenetic enzymes including HDAC3 and p300, further emphasizing the complexity of this pathway (Sun et al., 2025). In traumatic brain injury (TBI), a distinct mechanism has been identified whereby lactylation of Tufm, a key mitophagy regulator, impedes its interaction with mitochondrial proteins, suppressing mitophagy and promoting mitochondria-mediated neuronal apoptosis. Notably, genetic ablation of Tufm lactylation or therapeutic interventions such as mild hypothermia can restore mitophagic flux and reduce neuronal death, underscoring the pathophysiological relevance of lactylation in neuronal fate determination (Weng et al., 2025). Similarly, in the setting of subarachnoid hemorrhage, p300-mediated lactylation of β-arrestin1 (ARRB1) upregulates S100A9, facilitating mitochondrial dysfunction and neuronal apoptosis, while inhibition of this pathway mitigates injury (Mi et al., 2025). Moreover, in intracerebral hemorrhage, lactylation at the p53 promoter—driven by lactate dehydrogenase A (LDHA)—enhances p53 transcription, thereby promoting neuronal apoptosis, with LDHA knockdown reversing these effects (Zhang et al., 2025). Collectively, these findings demonstrate that lactylation dynamically modulates the transcriptional landscape of apoptosis-related genes and mitochondrial pathways, positioning it as a critical molecular switch in the regulation of neuronal survival and death following brain injury.

#### 2.4.2 Lactylation and mitochondrial dysfunction

Accumulating evidence demonstrates that lactylation serves as a critical regulator of mitochondrial function, particularly in the context of neuronal injury following stroke. Accumulating evidence demonstrates that elevated lactate levels after ischemic or hypoxic insults not only reflect metabolic stress but also directly influence mitochondrial homeostasis through protein lactylation. This modification has been shown to disrupt mitochondrial membrane potential, impair energy production, and exacerbate oxidative stress, thereby contributing to neuronal damage. Specific mitochondrial metabolic enzymes, such as malate dehydrogenase 2 (MDH2) and citrate synthase, have been identified as key substrates for lactylation. Their modification directly alters the tricarboxylic acid (TCA) cycle flux, thereby linking metabolic cues to mitochondrial bioenergetics (Tang et al., 2025). For example, in models of subarachnoid hemorrhage, increased lactate and lactylation of β-arrestin1 (ARRB1) in neurons led to mitochondrial respiratory dysfunction, reduced ATP synthesis, and enhanced neuronal apoptosis, effects that were mediated through the upregulation of S100A9 and could be mitigated by targeting the lactylation process (Mi et al., 2025). Similarly, global proteomic analyses in cerebral ischemia–reperfusion injury revealed widespread lysine lactylation of mitochondrial proteins, including those involved in the Ca2 + signaling pathway, ultimately affecting the mitochondrial apoptosis cascade and mediating neuronal death (Yao et al., 2023). Furthermore, studies in other organ systems reinforce these findings; for instance, lactylation of mitochondrial enzymes such as aldehyde dehydrogenase 2 (ALDH2) and citrate synthase has been shown to aggravate mitochondrial dysfunction by impeding mitophagy and activating inflammasome pathways, respectively, underscoring the conserved nature of this mechanism across tissues (Li et al., 2025; Yu et al., 2025). In the context of stroke, lactylation-induced mitochondrial dysfunction manifests as loss of membrane potential, impaired oxidative phosphorylation, and increased reactive oxygen species, all of which synergistically drive neuronal injury and PSF. Collectively, these studies highlight lactylation as a key mediator linking metabolic disturbances to mitochondrial dysfunction and neuronal damage, suggesting that targeting lactylation or its regulatory enzymes may offer novel therapeutic avenues for mitigating mitochondrial impairment, restoring energy supply, and potentially alleviating the fatigue symptoms associated with stroke recovery (Yao et al., 2023; Mi et al., 2025; Li et al., 2025; Yu et al., 2025). Synthesizing the upstream mechanisms of neuronal apoptosis and mitochondrial dysfunction, it becomes evident how these cellular pathologies culminate in the clinical syndrome of fatigue. The transition from acute ischemic injury to chronic PSF is not merely a consequence of structural damage but a result of sustained metabolic-epigenetic reprogramming. Specifically, the widespread lactylation induced by the initial ischemic insult locks neurons and glia into a maladaptive state. Neuronal injury and energy metabolism disorders are thus not isolated events but are integrally linked through lactylation networks to the persistent exhaustion experienced by patients. Following a stroke, disrupted cerebral blood flow leads to hypoxia and impaired glucose utilization, resulting in altered lactate production and accumulation. Lactylation, a post-translational modification of proteins by lactate, can influence gene expression and cellular function in neurons and glial cells. This modification affects mitochondrial activity and energy homeostasis, potentially exacerbating neuronal dysfunction and fatigue symptoms. Thus, the interplay between lactylation and metabolic disturbances provides a mechanistic link to the development and persistence of PSF.

### 2.5 Lactylation, neuroinflammation, and glial cell response

The extensive neuronal apoptosis and tissue injury described above are not terminal events; rather, they serve as potent triggers for a secondary wave of pathology: neuroinflammation. Dying neurons release danger signals that activate resident glial cells and recruit peripheral immune cells. In this inflammatory milieu, lactylation emerges as a critical epigenetic modulator, reshaping the functional phenotype of microglia and astrocytes to perpetuate the cycle of injury and fatigue.

#### 2.5.1 The role of lactylation in neuroinflammation

Following cerebral ischemic events, histone lactylation dynamically modulates the expression of key inflammatory cytokines, thereby influencing both the intensity and duration of the neuroinflammatory response. For instance, in models of acute ischemic stroke, lactate accumulation leads to increased histone H3K9 lactylation in microglia, which subsequently activates the transcription of glycolytic and pro-inflammatory genes such as Ldha and Hif-1α, exacerbating neuroinflammation. Notably, the regulatory protein SMEK1 was shown to modulate this process, where its deficiency in microglia enhanced lactate production and histone lactylation, while overexpression ameliorated neuroinflammation and improved neurological recovery, highlighting the therapeutic potential of targeting histone lactylation pathways (Si et al., 2024). Parallel findings in astrocytes reveal that histone H3K18 lactylation upregulates the expression of NSUN2, a methyltransferase that promotes m5C-dependent inflammatory gene expression, further amplifying astrocyte-driven neuroinflammation in ischemia–reperfusion injury. Pharmacological inhibition of lactate production or knockdown of NSUN2 attenuated the release of pro-inflammatory cytokines and reduced cerebral injury, underscoring the central role of the lactate-H3K18la-NSUN2 axis in mediating post-stroke neuroinflammation (Wang et al., 2025). Beyond stroke, studies in neurodegenerative and neuroinflammatory models, such as Parkinson’s disease and chronic fatigue syndrome, corroborate that glycolysis-derived lactate drives histone lactylation, which in turn promotes the transcription of inflammatory mediators through pathways like NF-κB and NOD2, intensifying neuroinflammatory cascades (Qin et al., 2025; Sun et al., 2025). Additionally, in bilirubin encephalopathy models, H3K18 lactylation has been shown to promote NOD2 expression and activate downstream MAPK and NF-κB pathways in astrocytes, mediating inflammatory responses (Li et al., 2025). Collectively, these findings delineate a mechanistic framework wherein lactylation acts as an epigenetic bridge linking metabolic reprogramming to the sustained activation of inflammatory genes, thus shaping the neuroinflammatory milieu after stroke and identifying lactylation as a promising target for therapeutic intervention.

#### 2.5.2 Glial cell activation and neuroenvironmental remodeling after stroke

Astrocytes and other glial cells play a central role in the neuroinflammatory response and glial cell activation following stroke, contributing to exacerbated neuronal injury and the development of PSF. Histopathological and immunohistochemical analyses of brain tissue from stroke patients reveal that both neurons and glial cells, particularly in the ischemic focus, are significantly affected by ischemic injury. The presence of extensive neuroinflammation is characterized by a complex infiltration of immune cells, including neutrophils, lymphocytes, plasma cells, and especially macrophages (Jian et al., 2019), which are most abundant in both the ischemic core and penumbra. This cellular milieu indicates robust glial activation, with astrocytes playing a central role in mediating the inflammatory response. The disruption of the blood–brain barrier, evidenced by endothelial discontinuities and perivascular edema (Bani-Sadr et al., 2023), further facilitates the infiltration of peripheral immune cells, amplifying local inflammation. The persistent activation of glial cells not only exacerbates neuronal damage via the release of cytotoxic mediators but also contributes to the maladaptive remodeling of the neural microenvironment (Nagy et al., 2020). Such changes are closely linked to the persistent neurological deficits and fatigue observed in stroke survivors. The interindividual variability in neuroinflammatory intensity, as observed in patient tissue analyses, suggests that the degree of glial cell activation may be influenced by factors such as comorbidities, age, and prior treatment, further complicating the neuroenvironmental remodeling process after stroke (Păun et al., 2023).

#### 2.5.3 Bidirectional regulation of lactylation and microenvironmental repair

Acting as a pivotal molecular bridge between cellular metabolism and epigenetic regulation, lactylation exerts a bidirectional influence on the tissue microenvironment. On one hand, lactylation can promote inflammation by modulating the expression of genes and signaling pathways involved in immune responses and cellular stress, thereby contributing to pathological processes such as chronic inflammation and immune evasion in disease states (Chen et al., 2025; Zhang et al., 2025). For instance, in the tumor microenvironment, lactylation of histones and non-histone proteins has been shown to activate genes associated with immunosuppressive phenotypes, such as Arg1 in M2 macrophages, which not only supports tumor growth but also fosters a pro-inflammatory, immunosuppressive milieu (Al-Malsi et al., 2025). Conversely, lactylation also plays a crucial role in tissue repair and regeneration by enhancing the transcriptional accessibility of repair-related genes. This duality is exemplified in regenerative processes such as cartilage repair, where increased histone lactylation under hypoxic and glycolytic conditions facilitates the activation of chondrogenic genes and matrix deposition, ultimately promoting effective tissue regeneration (Yang et al., 2025). Furthermore, the dynamic interplay between lactylation and its modifying enzymes orchestrates a finely tuned regulatory network that responds to microenvironmental cues, balancing inflammatory responses with reparative mechanisms (Chen et al., 2025). Thus, lactylation not only mediates inflammatory signaling but also actively participates in neuroregeneration and microenvironmental restoration by modulating the expression of key repair proteins. This bidirectional regulatory capacity underscores the therapeutic potential of targeting lactylation pathways to both limit deleterious inflammation and enhance endogenous repair processes following neurological injuries such as stroke.

### 2.6 Lactylation and post-stroke energy metabolism disorders

#### 2.6.1 The impact of lactylation on glycolysis and energy supply

Recent mechanistic studies have illuminated that lactylation can directly modify key glycolytic enzymes, thereby altering their function and the overall glycolytic flux. Glycolysis serves as the primary pathway for rapid ATP generation in brain tissue, especially under ischemic conditions where oxygen supply is compromised. Recent studies have illuminated that lactylation can directly modify key glycolytic enzymes, thereby altering their function and the overall glycolytic flux. For example, comprehensive lactylome analyses have demonstrated that virtually all enzymes involved in glycolysis and the tricarboxylic acid cycle can undergo lactylation, suggesting a pervasive regulatory mechanism that modulates metabolic adaptation in response to increased energy demands or pathological stress (Fan et al., 2025). This modification not only affects enzyme activity but also links metabolic status to gene expression through the lactylation of histones, further influencing the transcription of glycolysis-related genes (Xiong et al., 2025; Gaur et al., 2025). In neurological disorders, including acute ischemic stroke, lactylation has been implicated in the regulation of energy metabolism, where elevated lactate levels and increased glycolytic activity are observed in affected brain regions (Gu et al., 2025; Chen et al., 2025). The positive feedback loop between glycolytic flux, lactate production, and histone lactylation ensures a sustained supply of metabolic intermediates and ATP, which is crucial for neuronal survival and functional recovery after injury (Xiong et al., 2025). Moreover, lactylation may also influence the fate of non-histone proteins involved in metabolic control, further fine-tuning cellular energy responses (Fan et al., 2025). These findings collectively highlight lactylation as a key molecular bridge linking metabolic reprogramming to energy supply in the post-stroke brain, offering new perspectives for therapeutic intervention aimed at enhancing metabolic resilience and recovery following cerebral ischemia (Gu et al., 2025; Chen et al., 2025). Ultimately, the failure to restore efficient ATP production due to aberrant lactylation directly fuels the physical and cognitive exhaustion that characterizes PSF.

#### 2.6.2 Lactylation and oxidative stress response

The interplay between lactylation and oxidative stress is complex and multifaceted, impacting the expression and function of various antioxidant enzymes. The interplay between lactylation and oxidative stress is multifaceted. On one hand, increased lactate production under stress or ischemic conditions can lead to enhanced protein lactylation, which in turn modulates the transcription of genes involved in antioxidant defense. For instance, lactylation of mitochondrial proteins, such as malate dehydrogenase 2 (MDH2), has been shown to enhance NADPH generation, thus reducing reactive oxygen species (ROS) and conferring oxidative stress resistance in cells (Tang et al., 2025). Similarly, in the context of vascular endothelial cells exposed to oxidative stress, lactylation of key metabolic enzymes like lactate dehydrogenase A (LDHA) appears to support cell survival by promoting glycolytic flux and mitigating oxidative damage, suggesting that lactylation facilitates metabolic adaptation to stress (Wu et al., 2025). Conversely, there are also situations where excessive lactylation contributes to oxidative stress. For example, elevated histone lactylation (e.g., H4K12la) has been implicated in the activation of the FOXO1/PGC-1α pathway, leading to increased mitochondrial oxidative stress and tissue injury in models of diabetes-induced cognitive impairment (Yang et al., 2025). Moreover, environmental toxins such as nickel have been shown to induce histone H3 lactylation, resulting in ROS accumulation and DNA damage, thereby linking lactylation directly to oxidative injury (Xie et al., 2025). The modulation of antioxidant enzyme expression by lactylation is further supported by studies in inflammatory and metabolic disease models, where interventions that suppress lactylation (e.g., by pharmacological agents or genetic manipulation) can alleviate oxidative stress and tissue damage (Xu et al., 2024; Ma et al., 2024). Collectively, these findings underscore the dualistic role of lactylation in regulating oxidative stress after stroke, either by upregulating antioxidant defenses or by exacerbating oxidative injury, depending on the cellular context and the balance of metabolic signals. This highlights the therapeutic potential of targeting lactylation pathways to modulate oxidative stress responses in stroke and related neurological disorders. By mitigating oxidative damage, these interventions hold the potential to break the vicious cycle of inflammation and cellular fatigue, thereby alleviating the persistent symptoms of PSF.

#### 2.6.3 Mechanistic link between energy metabolism disorders and fatigue symptoms

Energy metabolism disorders are central to the development of PSF, with impaired energy supply directly contributing to the manifestation of fatigue symptoms. The brain and skeletal muscles depend on tightly regulated metabolic processes to meet their substantial energy demands, particularly during recovery from injury such as stroke. Disruption in these processes, whether due to mitochondrial dysfunction, impaired glycolysis, or defective substrate utilization, leads to an energy deficit that is closely associated with the onset and persistence of fatigue. Notably, lactate, long regarded as a mere byproduct of anaerobic metabolism and a marker of muscle fatigue, has emerged as a key intermediary linking energy metabolism disturbances to fatigue. Under physiological conditions, lactate serves as an alternative energy substrate, especially for the brain, and participates in the Cori cycle by being converted back to glucose in the liver, thereby supporting systemic energy homeostasis (Kumar et al., 2025). However, in pathological states such as ischemic stroke, excessive lactate accumulation can exacerbate metabolic disturbances, contributing to tissue damage and worsening fatigue symptoms (Kumar et al., 2025). Moreover, recent research highlights the role of lactylation—a novel post-translational modification—in mediating the effects of lactate on cellular function and gene expression, suggesting a direct epigenetic link between metabolic stress and the regulation of fatigue-related pathways (Wang and Zhu, 2025). Mitochondrial dysfunction, frequently observed in post-stroke and chronic fatigue conditions, impairs ATP production and further promotes lactate accumulation, creating a vicious cycle that perpetuates energy deficits and fatigue (Van Campenhout et al., 2025; Mantle et al., 2024). Clinical studies in metabolic myopathies and neurometabolic disorders reinforce this connection, demonstrating that inadequate energy supply and disrupted lactate metabolism are consistently associated with increased fatigue, muscle weakness, and reduced exercise tolerance (Schirinzi et al., 2023; Angelini, 2024). Thus, lactate and its downstream effects serve as a critical bridge between energy metabolism disorders and the pathophysiology of PSF, underscoring the importance of targeting metabolic pathways and lactate signaling in therapeutic strategies aimed at alleviating fatigue symptoms in stroke survivors.

### 2.7 Research progress on lactylation as a potential therapeutic target for post-stroke fatigue

#### 2.7.1 Pharmacological studies of lactylation modulators

**Figure 3:**
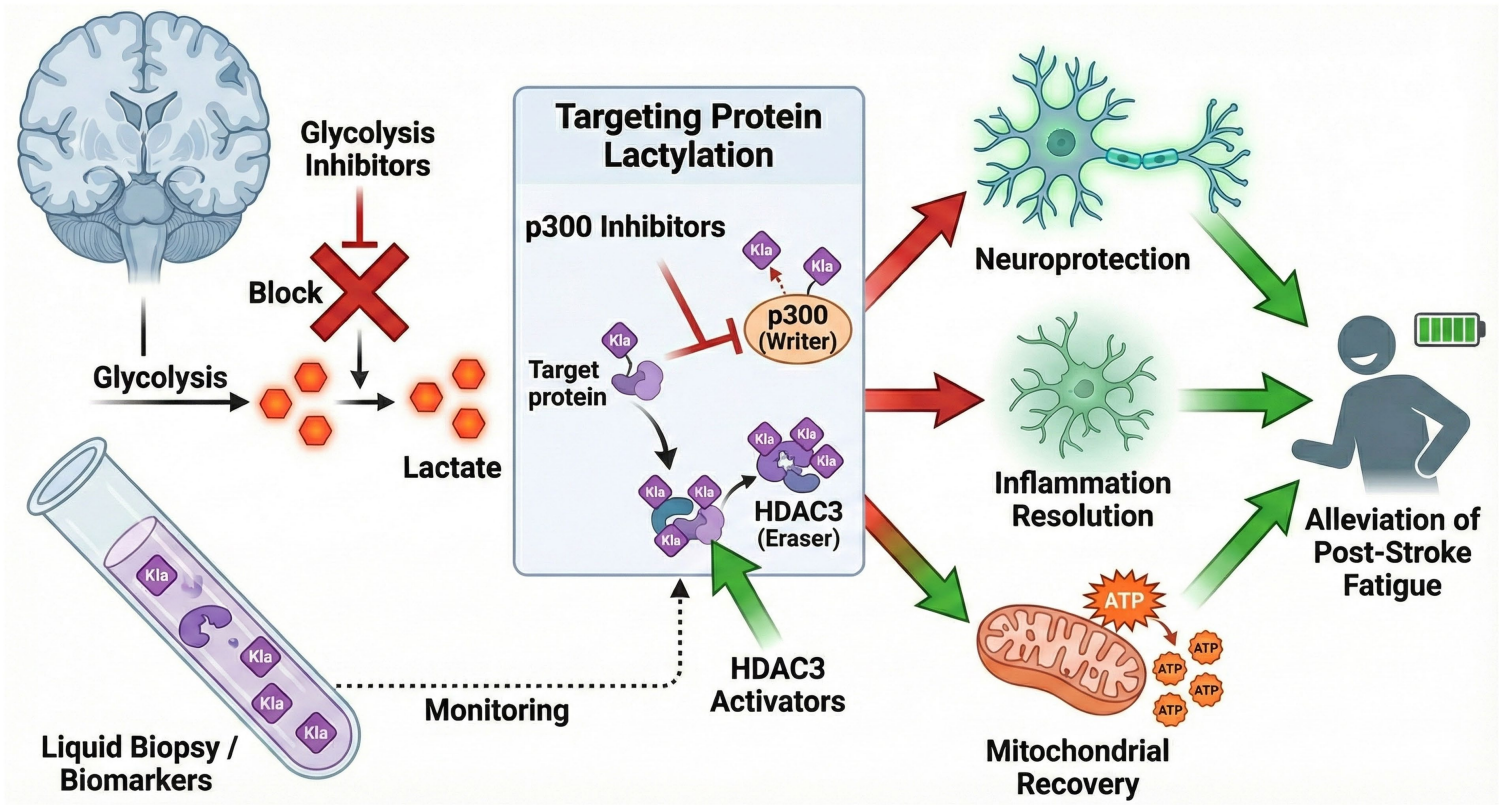
Therapeutic strategies targeting lactylation for post-stroke fatigue. Potential interventions targeting the lactylation pathway. (Left) Diagnosis: Liquid biopsy detecting lactylated protein biomarkers helps monitor disease progression. (Center) Pharmacological intervention: Targeting the “writer” (p300 inhibitors) or activating the “eraser” (HDAC3 activators) can reverse aberrant hyper-lactylation. (Right) Therapeutic outcomes: These interventions aim to reduce neuroinflammation, protect neurons against apoptosis, and restore mitochondrial ATP production, ultimately alleviating the symptoms of post-stroke fatigue.

Recent advances in the understanding of lactylation as a key post-translational modification have spurred interest in pharmacological agents that target this pathway, particularly in the context of neurological injury and PSF. Among the most promising are inhibitors of the histone acetyltransferase p300 and activators of class I histone deacetylases, such as HDAC3. These enzymes are central to the regulation of histone and non-histone protein lactylation, directly influencing gene transcription and cellular fate following cerebral ischemia and other forms of neural injury. For instance, studies have demonstrated that inhibition of p300 can reduce pathological histone lactylation, thereby attenuating neuronal apoptosis and improving neurological outcomes in animal models of ischemic stroke. Specifically, p300 has been shown to mediate lactylation of key transcriptional regulators such as MeCP2, with pharmacological inhibition resulting in increased infarct volume and worsened behavioral recovery (Sun et al., 2025). These findings are corroborated by broader research indicating that pharmacological targeting of lactylation—whether through direct modulation of lactyltransferase or delactylase activity—can influence disease outcomes not only in the CNS but also in cancer and inflammatory disorders, underscoring the therapeutic potential of this approach (Chen et al., 2025; Rho and Hay, 2025). In summary, the pharmacological manipulation of lactylation via p300 inhibitors and HDAC3 activators represents a promising avenue for the mitigation of post-stroke injury and fatigue, with preclinical data supporting their efficacy and laying the groundwork for future translational studies (Figure 3). In parallel with therapeutic development, the identification of lactylation-related biomarkers in cerebrospinal fluid or blood holds significant promise for the early prediction and monitoring of PSF. While lactate metabolism indices are already established prognostic markers in ischemic stroke (Jin et al., 2022), recent advances in oncology have further demonstrated the feasibility of detecting specific lactylated proteins in liquid biopsies as diagnostic biomarkers (Wang et al., 2024). Elevated levels of these modified proteins may reflect underlying metabolic and neuroinflammatory changes associated with stroke and subsequent fatigue development, offering a novel molecular window for precision diagnosis.

#### 2.7.2 Clinical translation prospects and challenges

The clinical translation of lactylation-targeted interventions for PSF presents both promising prospects and significant challenges. While preliminary studies suggest that modulating lactylation may alleviate fatigue symptoms, the safety and efficacy of such approaches remain to be fully established in human populations. Moreover, individual variability in metabolic responses and genetic backgrounds necessitates the development of personalized treatment strategies. Rigorous clinical trials are required to assess potential side effects, optimal dosing, and long-term outcomes. Addressing these issues will be crucial for the successful integration of lactylation-based therapies into routine clinical practice for PSF management.

## 3 Conclusion

Lactylation represents a paradigm shift in our understanding of the pathophysiology underlying Post-Stroke Fatigue. By acting as a dynamic “metabolic-epigenetic bridge,” this post-translational modification translates the acute metabolic stress of ischemia—specifically lactate accumulation—into sustained neuroinflammatory responses and neuronal energy deficits. Current evidence synthesizes a clear pathological trajectory: the hypoxia-induced surge in lactate fuels p300-mediated lactylation of histones and metabolic enzymes, which locks microglia into a pro-inflammatory phenotype and impairs mitochondrial bioenergetics in neurons. This sustained cellular dysfunction manifests clinically as the profound physical and cognitive exhaustion characteristic of PSF.

Looking forward, the identification of key regulatory nodes, such as the p300/HDAC3 enzymatic axis and specific lactylation sites on proteins like MeCP2, offers novel avenues for therapeutic intervention. Future research should prioritize the development of highly specific lactylation modulators and validate the utility of lactylated proteins as fluid biomarkers. Successfully translating these mechanistic insights into clinical practice holds the promise of breaking the vicious cycle of metabolic dysregulation and inflammation, ultimately improving the quality of life for stroke survivors suffering from fatigue.
